# Supplementary material for: Detection of latent brain states from baseline neural activity in the amygdala
Source: bioRxiv. 2024 Jun 14:2024.06.14.598974. Preprint. [Version 1] doi: 10.1101/2024.06.14.598974 (PMC11195171; doi:10.1101/2024.06.14.598974)
Supplement: Supplement 1 [file NIHPP2024.06.14.598974v1-supplement-1.pdf]

bioRxiv preprint doi: https://doi.org/10.1101/2024.06.14.598974; this version posted June 14, 2024. The copyright holder for this preprint (which was not certified by peer review) is the author/funder, who has granted bioRxiv a license to display the preprint in perpetuity. It is made available under aCC-BY 4.0 International license.

SUPPORTING INFORMATION

Baseline selection criteria

Baseline LFP was selected from a stable time window of the interstimulus interval (ISI) between two stimuli of the same type. The ISI was defined as the period occurring 200ms after stimulus offset and 200ms before stimulus onset. For each recording session, all ISI signals were trial averaged and the standard deviation for each timepoint was calculated. Baseline LFP for each trial was chosen by inspecting the trial-averaged ISI and determining a time window with low trial-wise variability.

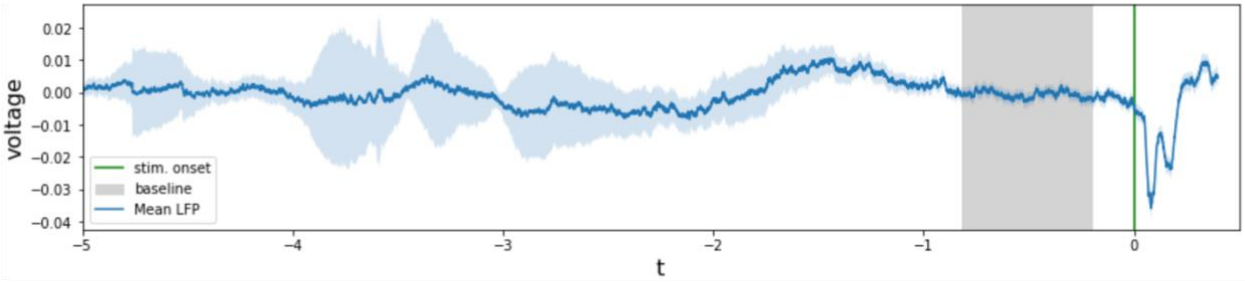

Figure S1. ***Selecting stable baseline trials.*** *An example of the baseline LFP selection criteria for a single recording session. The solid blue line is the trial-average of the signals during each ISI. The vertical green line is stimuli onset. The light blue shading is 2 st. dev. of the mean. The gray box is the stable time window chosen as the baseline.*

Linear SVM fails to discriminate context reliably

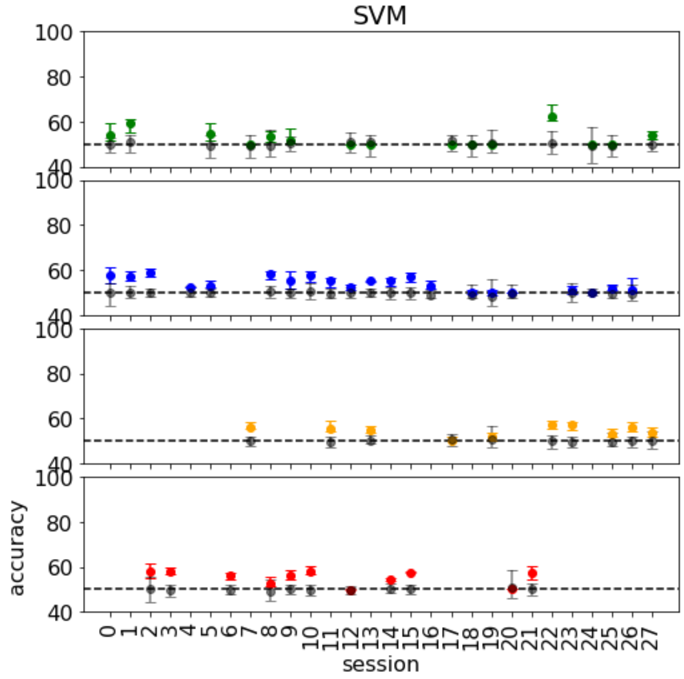

Figure S2. ***Decoding context fails with linear SVM.*** *Average accuracy was computed over 50 sample linear SVMs. Trials were randomly reassigned to the training, validation, and testing sets for each sample classifier using an 80-10-10 split. Accuracy results for all recording sessions using the CNN classifier. The 50% quantile of accuracy is represented by a dot, with vertical bars reporting the 10% and 90% quantiles. Colors indicate the nucleus in which the recording contacts were located. Gray bars indicate the null distribution obtained from bootstrapping.*

bioRxiv preprint doi: https://doi.org/10.1101/2024.06.14.598974; this version posted June 14, 2024. The copyright holder for this preprint (which was not certified by peer review) is the author/funder, who has granted bioRxiv a license to display the preprint in perpetuity. It is made available under aCC-BY 4.0 International license.

Spectrogram classification failed on SVM without using the kernel trick. This is likely because the time-frequency features are highly correlated and require a non-linear mapping to improve separability. Results from the linear SVM classification are shown in **Figure S2**. Average accuracy across nuclei and sessions rarely exceeds 60% and often is not statistically different from the null distribution. Overall, the linear SVM classification is less reliable than performance from both SVM with kernel trick and the CNN, both of which leverage non-linear transformations of the data before performing classification, indicating that the non-linear embedding is necessary for classification.

Classification accuracy depends on dataset size

Variability across recording sessions and nuclei is due, in part, to the number of recording electrodes present during recording sessions, which determines the total amount of data. Machine learning methods are notoriously data-hungry, and typically require a large amount of data to adequately learn a particular task. **Figure S3** show the classification accuracy as a function of the number of recording electrodes in a particular nucleus during a recording session. Results for all three subjects are shown. For both CNN and SVM, the accuracy of days with a single recording electrode is below 60% but quickly improves with the addition of more data. Both classifiers also show performance generally plateauing between 70-85% which can be achieved with 5 or more contacts present. This exploration explains why performance of the classifier on the central nucleus is (slightly) lower than other nuclei across subject. The relative size of the central amygdala compared to other nucleus is much smaller, limiting the total amount of electrodes that can be present in central amygdala in any given session. Nevertheless, we still see both SVM and CNN classifiers performing better than chance when data from more than one recording electrode is available.

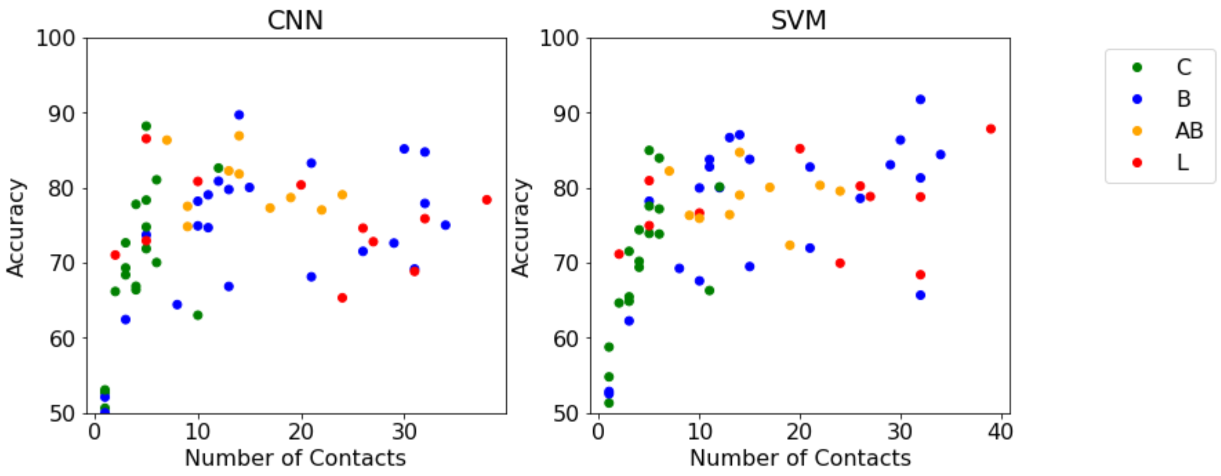

Figure S3. ***Decoding accuracy is a function of data availability (number of recording electrodes).*** Classification accuracy for one sample of CNN (left) and SVM (right). Each dot represents the accuracy of a single classifier trained on data from one session and nucleus.

bioRxiv preprint doi: https://doi.org/10.1101/2024.06.14.598974; this version posted June 14, 2024. The copyright holder for this preprint (which was not certified by peer review) is the author/funder, who has granted bioRxiv a license to display the preprint in perpetuity. It is made available under aCC-BY 4.0 International license.

Computational cost of CNN and SVM

Accuracy results in the main body of the text suggest that although SVM and CNN perform the same on average, the SVM classification exhibits less variability across the 50 realizations (evidenced by the narrower confidence intervals). Readers may be tempted to assume that SVM implementation should therefore be preferred over CNN. However, the choice in classifier may be better suited by the amount of data available. **Figure S4** shows the computational cost of implementing both CNN and SVM as a function of the number of recording electrodes. As mentioned above, the number of recording electrodes will strongly influence the amount of data in the training set. For small amount of data (<4 electrodes), SVM is the more efficient implementation. However, overall, SVM scale quadratically with the number of electrodes (as expected) while the CNN scales linearly. For moderate and large amounts of data, the CNN is more efficient.

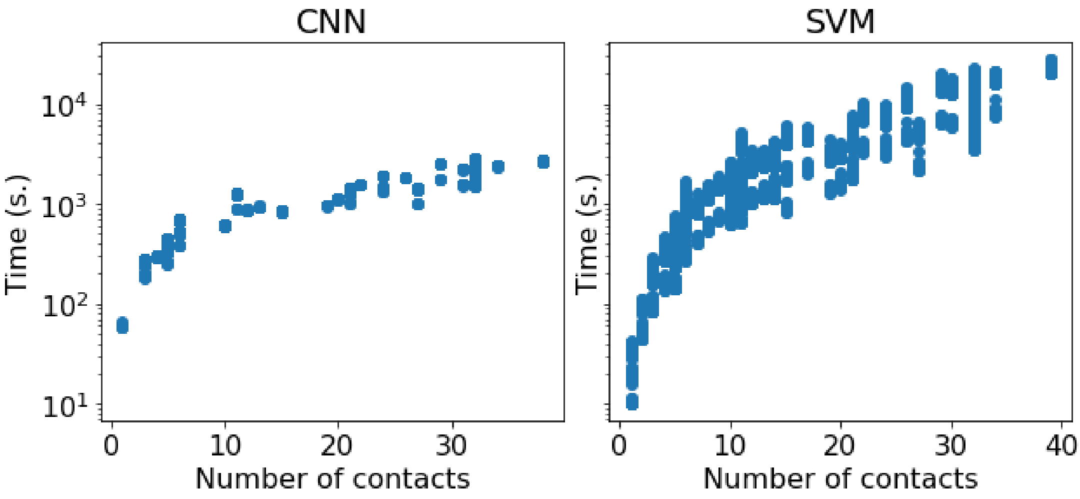

Figure S4. ***Computational costs of CNN and SVM.*** The computational time for training a single classifier for CNN (left) and SVM (right) as a function of the number of recording electrodes (which most influences training dataset size). Each dot represents a single realization of the classifier.

Classification performance decreases dramatically using raw time-series alone

The methodology presented in the main text uses trial spectrograms as the feature space for decoding. As a first step in our analysis, we tried traditional classification methods, like SVM, using the raw time series data. Though these methods were able to discriminate between airflow and grooming contexts, the accuracy results were little better than chance. **Figure S5** shows the accuracy of SVM decoding using the raw time series trials compared to spectrograms. Decoding accuracy noticeably improves across all subjects and nuclei when using time-frequency spectrograms (Monkey A: 10% increase, Monkey C: 18%, Monkey S: 20%)

bioRxiv preprint doi: https://doi.org/10.1101/2024.06.14.598974; this version posted June 14, 2024. The copyright holder for this preprint (which was not certified by peer review) is the author/funder, who has granted bioRxiv a license to display the preprint in perpetuity. It is made available under aCC-BY 4.0 International license.

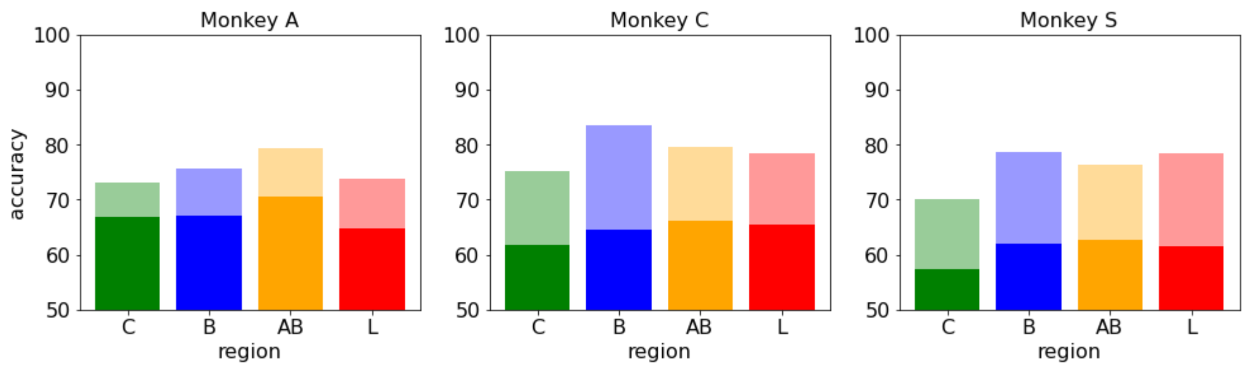

Figure S5. **Comparison of SVM using raw timeseries data over spectrogram.** SVM classification accuracy for each nucleus, averaged over all sessions for the same subjects (Monkey A (left), Monkey C (middle), Monkey S (right)). More saturated colors indicate the SVM accuracy using raw time series trials. SVM decoding accuracy using trial spectrograms (reported in the main text) is shown in more transparent color for comparison.
